# Lipid droplet biogenesis and COX-2 pathway activation are triggered by Barrett’s esophagus and adenocarcinoma, but not esophageal squamous cell carcinoma risk factors

**DOI:** 10.1038/s41598-020-80035-4

**Published:** 2021-01-13

**Authors:** N. Carrossini, N. Meireles Da Costa, E. Andrade-Barreto, V. P. L. Sousa, P. Nicolau-Neto, P. T. Souza-Santos, G. R. Mansur, L. Wernersbach, P. T. Bozza, J. P. B. Viola, Luis Felipe Ribeiro Pinto

**Affiliations:** 1 Molecular Carcinogenesis Program (PCM), Research Coordination (CPQ), Brazilian National Cancer Institute (INCA), Rua André Cavalcanti, 37-Centro, 6º andar, Rio de Janeiro, RJ CEP 20231-050 Brazil; 2 Laboratory of Immunopharmacology, Oswaldo Cruz Institute-FIOCRUZ, Av. Brasil 4365, Manguinhos, Rio de Janeiro, RJ CEP 21040-360 Brazil; 3 Endoscopy Section, Brazilian National Cancer Institute-INCA, Praça Cruz Vermelha, 23, 8º andar, Centro, Rio de Janeiro, RJ CEP 20230-130 Brazil; 4 Pathology Division, Brazilian National Cancer Institute-INCA, Rua Cordeiro da Graça, 156, Santo Cristo, Rio de Janeiro, RJ CEP 20220-400 Brazil; 5 Program of Immunology and Tumor Biology, Brazilian National Cancer Institute-INCA, Rua André Cavalcanti, 37, 5º andar, Centro, Rio de Janeiro, RJ CEP 20231-050 Brazil; 6 Biochemistry Department, Instituto de Biologia Roberto Alcântara Gomes, Universidade Do Estado Do Rio de Janeiro, Boulevard 28 de Setembro, 77-Maracanã, Rio de Janeiro, RJ CEP 20551-030 Brazil

**Keywords:** Cancer, Cell biology, Molecular biology, Oncology, Risk factors

## Abstract

Esophageal cancer (EC) is an aggressive disease, presenting two main histological subtypes: adenocarcinoma (EAC) and squamous cell carcinoma (ESCC). The two EC subtypes widely differ concerning virtually all factors. ESCC development is mainly associated with tobacco and alcohol abuse, whereas obesity and chronic gastroesophageal reflux disease (GERD) are important risk factors not only for EAC, but also for for Barrett’s esophagus (BE), an intestinal metaplasia that precedes EAC. Obesity triggers ectopic lipid droplets (LD) accumulation in non-adipose tissues. LD are organelles involved in cell metabolism, signaling, proliferation and production of inflammatory mediators. Therefore, the aim of this work was to investigate LD occurrence and role in EC. This study shows progressive LD levels increase along EAC development, in esophageal samples from non-obese through obese individuals, as well as BE, and EAC patients, whereas no significant changes were observed in ESCC samples, when compared to non-tumor samples. Additionally, in order to mimic BE and EAC risk factors exposure, a non-tumor esophageal cell line was incubated with oleic acid (OA) and acidified medium and/or deoxycholic acid (DCA), revealing a significant increment in LD amount as well as in *COX-2* and *CXCL-8* expression, and in IL-8 secretion. Further, COX-2 expression and LD amount presented a significant positive correlation and were detected co-localized in EAC, but not in ESCC, suggesting that LD may be the site for eicosanoid production in EAC. In conclusion, this study shows that obesity, and BE- and EAC-associated inflammatory stimuli result in a gradual increase of LD, that may be responsible for orchestrating inflammatory mediators’ production and/or action, thus contributing to BE and EAC genesis and progression.

## Introduction

Esophageal cancer (EC) is the sixth leading cause of cancer-related death worldwide^1^, with 5-year survival rate ranging from 15 to 25% of patients^2^. There are two main EC histological subtypes: squamous cell carcinoma (ESCC) and adenocarcinoma (EAC). Although ESCC represents the predominant histological subtype of esophageal tumors worldwide, the incidence of EAC has greatly increased in the last decades^3^. The two EC subtypes widely differ concerning the etiological factors, incidence, geographical distribution, molecular alterations, among others. ESCC is mainly associated with tobacco and alcohol abuse^3^, whereas EAC is usually preceded by Barrett’s Esophagus (BE), an intestinal metaplasia of the distal esophagus in which squamous cells are replaced by a columnar epithelium in response to the damage caused by chronic and severe gastroesophageal reflux disease (GERD), an EAC risk factor together with obesity^4^. Thus, EAC development is represented by a sequence of consecutive changes, from esophagitis to BE and, finally into an adenocarcinoma, with inflammation as a driving force of the neoplastic transformation^5^.

Lipid droplets (LD) are lipid-rich cytoplasmic organelles formed by a neutral lipid core surrounded by a monolayer of phospholipids and coated proteins, such as the perilipin proteins (PLIN1-5). In response to metabolic signals, mobilized fatty acids (FAs) derived from stored neutral lipids inside LD are used for a variety of cell functions, including energy production via ß-oxidation, membrane biogenesis for cell growth, protein modification, signaling, and even secretion within lipoproteins^6^. In the past, LD were mainly associated with lipid accumulation and transport, but nowadays they are recognized as dynamic and ubiquitous organelles that play crucial roles in cellular signaling and production of inflammatory mediators^7^.

Lately, the role of LD in carcinogenesis has been brought to attention. Increased levels of LD and its structural proteins have been observed in some tumors such as colorectal^8^, hepatocellular^9^, Burkitt lymphoma^10^, and lung adenocarcinoma^11^, among others. PGE_2_ synthesis in colon cancer cells occur essentially in LDs, suggesting that these organelles may be implicated with the development of inflammatory responses and pathogenesis of colon adenocarcinoma^8^. Furthermore, several proteins already detected in LD, such as phosphoinositide-3-Kinase (PI3K), extracellular signal-regulated kinase (ERK) 1, ERK2, protein kinase C (PKC) and caveolin^12,13^, play established roles in inflammatory process and carcinogenesis, reinforcing the link between LD, inflammation, and tumorigenesis.

Considering the importance of inflammation for the development of esophageal tumors and the potential involvement of LD in inflammatory processes and tumorigenesis, the aim of this work was to investigate the occurrence of LD in esophageal tumors, and its potential contribution to their genesis and/or progression.

## Results

### LD amount is increased along EAC development

**Figure 1:**
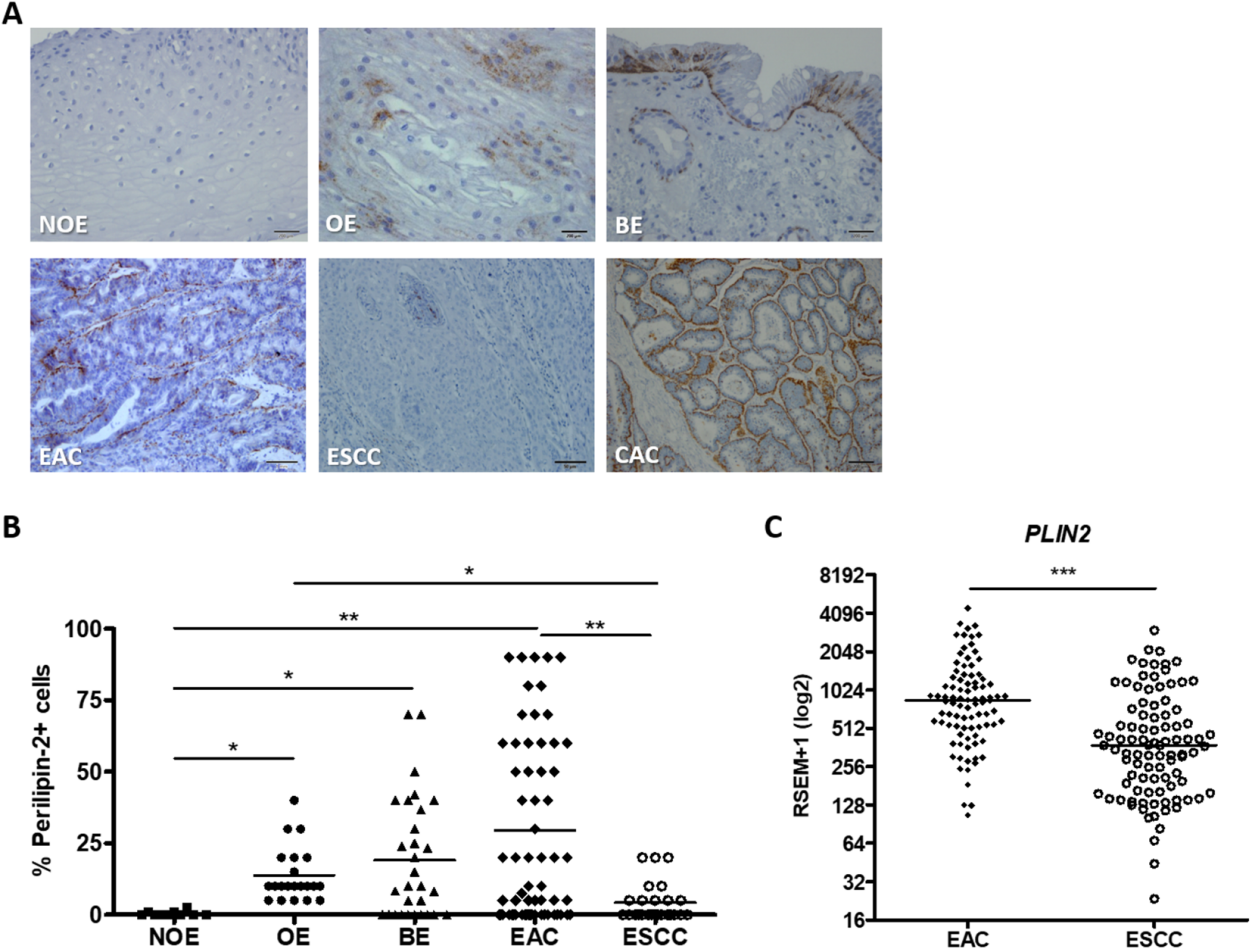
Perilipin-2 expression pattern in esophageal samples. (**A**) Representative immunohistochemistry micrographs of esophageal samples from non-obese individuals (NOE; n = 10), obese individuals (OE; n = 21), Barrett’s esophagus (BE; n = 30), esophageal adenocarcinoma (EAC; n = 64) and esophageal squamous cell carcinoma (ESCC; n = 25) patients stained for Perilipin-2. Colon adenocarcinoma (CAC) samples were used as positive control for Perilipin-2 staining. (**B**) Graphical representation of the esophageal samples’ Perilipin-2 staining score. (**C**) PLIN-2 mRNA expression evaluated in 88 EAC and 95 ESCC samples, from patients whose data is available at TCGA database. Gene expression pattern is evaluated from RNA-seq data, and represented as RSEM-1 units *p < 0.05; **p < 0.005; ***p < 0.0005.

Initially, we evaluated the presence of LD along EAC evolution and in ESCC, through perilipin-2 (PLIN2) expression analysis. PLIN2 is a structural protein present in LD’s surface^14^, and commonly employed to investigate LD presence^9^. Figure 1A,B show that there was a gradual increase in PLIN2 immunohistochemistry positive staining along EAC evolution. So, PLIN2 was not detected in any of the ten non-obese esophageal (NOE) samples, whereas a poor and diffuse perilipin-2 staining was observed in all 21 esophageal samples from obese patients (OE, with a median of 13.8% positive cells). PLIN2 staining was strong in 70% of 30 BE samples (with a median of 20.6% positive cells) and in 65% of 64 EAC samples (with a median of 29.5% positive cells). The pattern of PLIN2 staining observed in EAC samples was similar to that of colon adenocarcinoma samples, used as positive control (Fig. 1A). Differently from EAC, ESCC samples displayed discrete and diffuse expression of PLIN2, with only 24% of 25 samples presenting positive staining, with a median of 4.2% positive cells (Fig. 1A,B).

Complementary, to validate the higher number of LD in EAC than in ESCC, 88 EAC and 95 ESCC samples, with molecular and clinico-pathological data deposited in TCGA database, had their *PLIN2* expression re-analyzed. This analysis showed a significant higher expression of this gene in EAC, when compared to ESCC samples (Fig. 1C). *PLIN2* encodes the protein PLIN2 and is involved in LD synthesis^15^.

The socio-demographic and clinicopathological data of the patients evaluated are described in Supplementary Tables 1 and 3. Association analysis between BE, EAC and ESCC patients clinico-pathological data and PLIN2 protein expression was performed revealing a statistically significant association between PLIN2 expression and the presence of dysplasia in BE patients (Supplementary Table 2). Additionally, association analysis between clinicopathological features of EAC and ESCC patients from TCGA database and *PLIN2* gene expression was also performed, and a significant association between *PLIN2* expression and gender was observed in ESCC patients (Supplementary Table 4).

Together, these results demonstrate that LD are more abundant in EAC than in ESCC tumors and this phenomenon seems to be regulated along EAC development.

### EAC and BE, but not ESCC risk factors associated stimuli significantly increment LD biogenesis

Next, we investigated whether BE and EAC associated risk factors would impact on the induction of LD formation in vitro. In this way, the normal immortalized esophageal cell line, Het1a, was incubated with 1 µM, 2 µM or 5 µM of oleic acid (OA), or exposed to increasing number of pulses of either acidified cell culture medium (pH 5.0), or Deoxycholic Acid (DCA), or both combined. Cells were, then, stained with Oil Red O (ORO), and positively stained cells were quantified. OA is the most abundant free fatty acid present in our diet^16^ and is related to obesity, whereas DCA is a bile acid present in the content of gastro-esophageal reflux^17^ and, together with acidified culture medium, simulates this risk factor.

**Figure 2:**
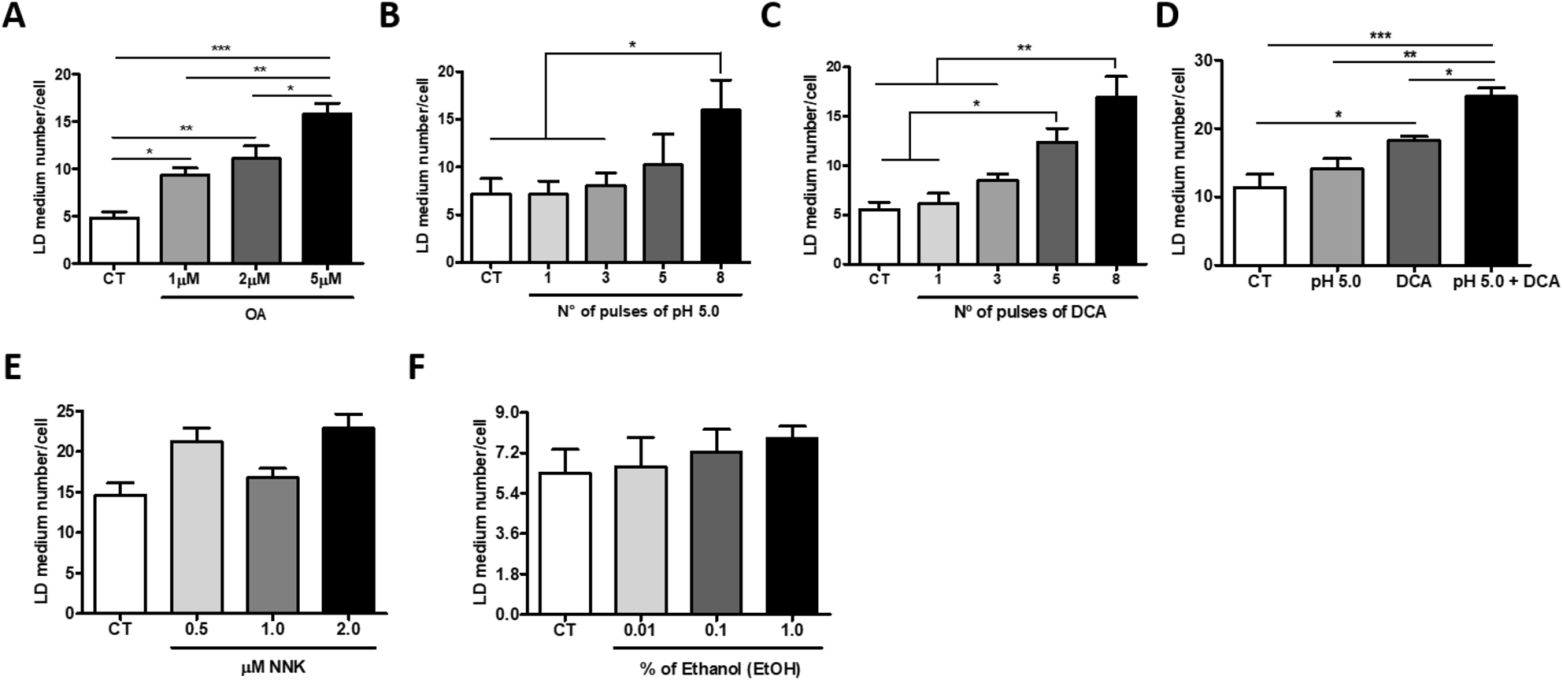
Lipid droplets (LD) number evaluation in the normal immortalized esophageal cell line, Het1a, upon exposure to esophageal tumors risk factors associated stimuli. LD quantification assessed by Oil Red O (ORO) staining following Het1a incubation with 1 µM, 2 µM or 5 µM of Oleic Acid (OA) for 24 h (**A**); Het1a stimulation with increasing number of acidified medium (pH5.0) (**B**), or 200 µM Deoxycholic Acid (DCA) (**C**) 3 min pulses, or the combination of both (**D**); Het1a treated with increasing concentrations of NNK (Nicotine-derived nitrosamine ketone) (**E**) and ethanol (**F**) for 24 h. Experiments were performed in triplicate *p < 0.05; **p < 0.005; ***p < 0.0005.

Figure 2A shows that the incubation of Het1a cells with 1 µM, 2 µM and 5 µM of OA for 24 h resulted in 1.9-, 2.3- and 3.3-fold increase in LD number, respectively, when compared to control cells. Exposure of Het1a cells to eight pulses (3 min each) of acidified medium (pH 5.0) also resulted in a significant increase of about twofold in the number of LD, when compared to non-exposed cells (Fig. 2B). Similarly, Het1a cells exposed to 5 and 8 pulses of 200 μM DCA presented a significant increase of 2.2- and 3.0-fold in the number of LD, respectively, when compared to control cells (Fig. 2C). Further, the combination of the two stimuli, 3 pulses of 200 μM DCA in acidified medium (pH5.0), conferred a greater increase in the number of LD, when compared to the effect of either factor alone (Fig. 2D). Additionally, the incubation of Het1a cells with OA (5 µM for 24 h) significantly induced *FASN* (*Fatty Acid Synthase*) and *CDX2* (*Caudal Homeobox Gene 2*) expression, and their exposure to 5 pulses of acidified medium (pH 5.0) containing 200 μM DCA, significantly induced *PLIN2, FASN* and *CDX2* expression, when compared to control cells (Supplementary Fig. 1). *FASN* encodes the enzyme fatty acid synthase that is involved in LD synthesis^18^. *CDX2* is a marker of intestinal development, and is involved with the pathogenesis of BE^19^.

Further, in order to verify whether the induction of LD formation is an event exclusively triggered by the exposure to EAC risk factors associated stimuli, the simulation of ESCC risk factors in vitro was also performed. To this aim, Het1a cells were treated with increasing concentrations of NNK (Nicotine-derived nitrosamine ketone), a key tobacco-specific nitrosamine^20^, in order to simulate tobacco smoking, and ethanol, for 24 h, in order to simulate alcohol consumption, and positively ORO stained cells were quantified. Nevertheless, contrary to BE and EAC risk factors associated-stimuli, neither NNK nor ethanol treatment, or the combination of both, led to significant changes in LD number in Het1a cells, when compared to non-treated cells (Fig. 2E,F). Further, EAC e ESCC derived cell lines, OE-33 and OE-21, respectively, were incubated with OA (5 µM for 24 h) and acidified DCA (5 pulses of 200 μM DCA in acidified medium—pH5.0) and a significant induction in LD number was detected only in the EAC derived cell line, OE-33 (Supplementary Fig. 2).

Thus, collectively, these data indicate that the risk factors associated with EAC development, but not those related to ESCC, are capable of inducing LD formation.

### Greater LD abundance in EAC cell line is accompanied by increased expression and secretion of inflammatory mediators

**Figure 3:**
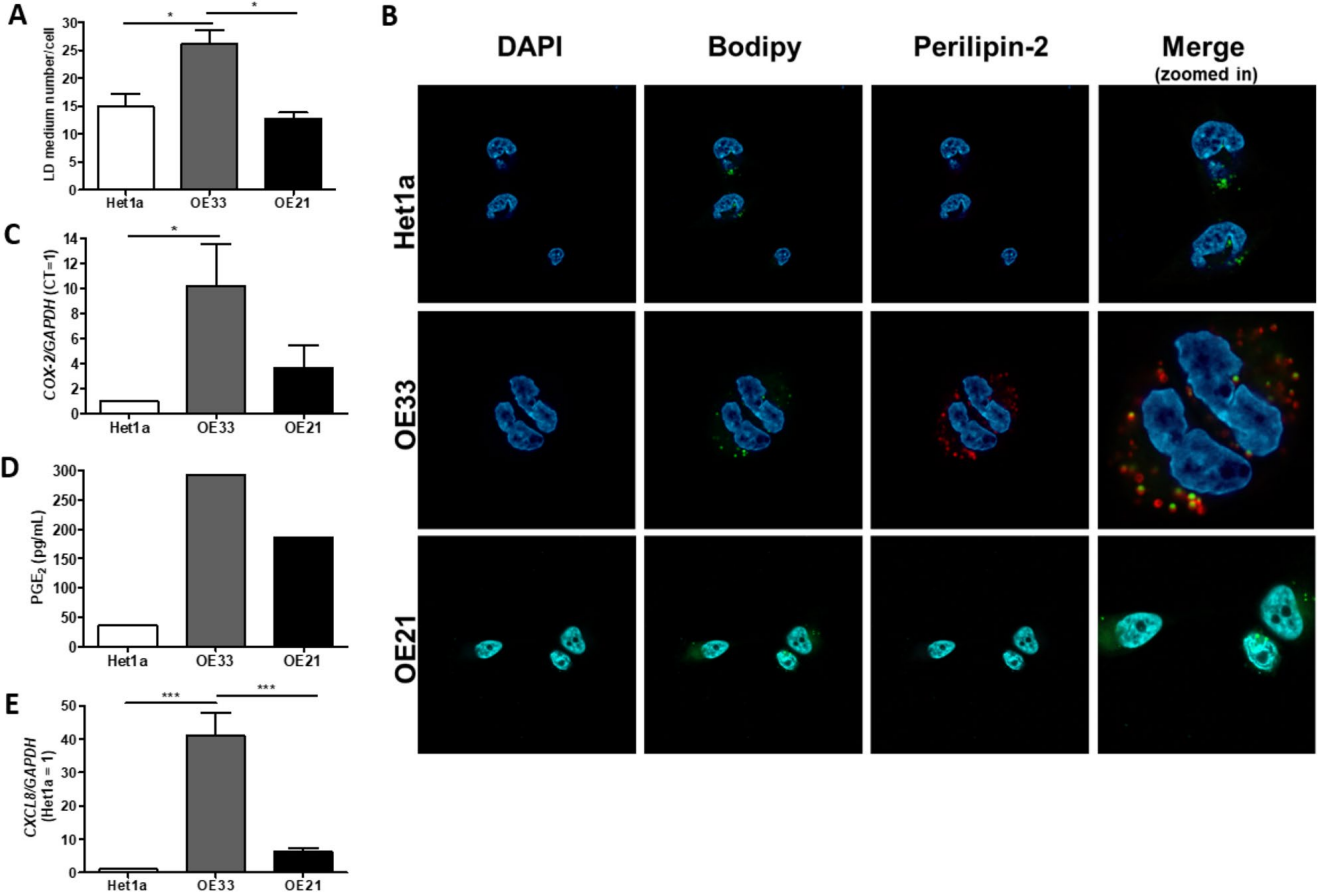
Evaluation of Lipid Droplets (LD) number and inflammatory mediators expression and/or secretion in esophageal cell lines. (**A**) LD quantification assessed by Oil Red O (ORO) staining in the normal immortalized esophageal cell line, Het1a, and in the esophageal adenocarcinoma (OE33) and squamous cell carcinoma (OE21) cell lines. (**B**) LD (by using Bodipy) and Perilipin-2 staining in Het1a, OE33 e OE21 cell lines. Blue color represents DAPI staining; Green color represents Bodipy staining; Red color represents Perilipin-2 staining. (**C**) COX-2 and (**E**) CXCL-8 mRNA expression, assessed by RT-qPCR, and (**D**) PGE2 secretion, assessed by ELISA, evaluated in Het1a, OE33 e OE21 cell lines. Experiments were performed in triplicate ***p < 0.0005.

The difference observed in the presence of LD between BE and EAC to ESCC tumors was further investigated. To this, EAC and ESCC cell lines, OE-33 and OE-21, respectively, as well as the normal immortalized esophageal cell line, Het1a, were employed. These cells were stained with ORO and positively stained cells were quantified. Figure 3A shows that OE-33 cell line presented a statistically significant higher LD number, when compared to OE-21 or Het1a cell lines. In addition, the presence of PLIN2 protein was also evaluated in the same cell lines, by immunofluorescence. Figure 3B demonstrates that only OE-33 cells were PLIN2 positive, and this protein was co-localized with Bodipy. Thus, the number of LD in the cell lines investigated reflects the panorama observed in the human esophageal tumor samples.

Further, considering the intimate relationship between LD and inflammation, the following step was to investigate the expression of molecules involved with inflammatory processes and that have already been associated with the development and/or progression of esophageal tumors^21,22^. Firstly, *COX-2* (*Cyclooxygenase-2*) expression and PGE-2 (Prostaglandin E2) production was assessed in Het1a, OE-33, and OE-21 cell lines, by RT-qPCR and ELISA, respectively. Figure 3C shows that whereas OE-33 cells present COX-2 mRNA levels almost 9.0-fold higher than those of the normal esophageal cells, OE-21 cells did not show any significant difference when compared to Het1a. Complementary, Fig. 3D shows that PGE2 production by OE-33 cells was 7.5- and 1.5-fold higher than that by Het1a and OE-21 cells, respectively. Next, *CXCL8* (*C–X–C Motif Chemokine Ligand 8*) mRNA levels were investigate in the same cell lines and pointed out a 40-fold incremented in CXCL8 mRNA expression in OE-33 cells, comparing to Het1a, whereas no significant difference was observed between OE-21 and Het1a (Fig. 3E). Hence, the greater number of LD detected in the EAC cell line, OE-33, is accompanied by the increased expression and secretion of inflammatory mediators.

### COX-2 is upregulated in early steps of BE and EAC evolution and is co-expressed with perilipin-2 in this tumor

**Figure 4:**
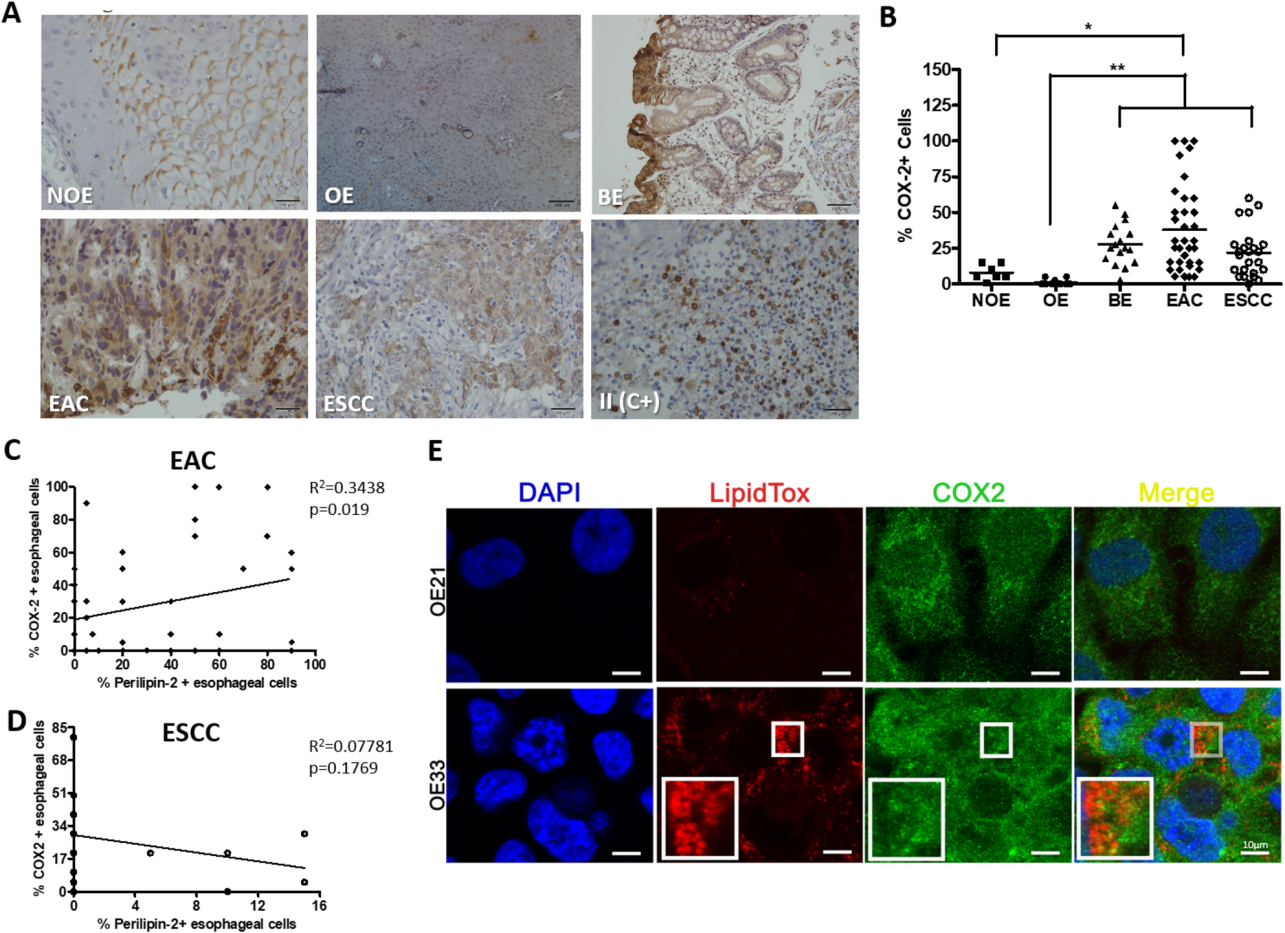
COX-2 expression pattern in esophageal samples and its relationship with Lipid Droplets (LD) presence. (**A**) Representative immunohistochemistry micrographs of esophageal samples from non-obese individuals (NOE; n = 10), obese individuals (OE; n = 21), Barrett’s esophagus (BE; n = 30), esophageal adenocarcinoma (EAC; n = 64) and esophageal squamous cell carcinoma (ESCC; n = 25) patients stained for Perilipin-2. The inflammatory infiltrate (II) was used as a positive control for COX-2 staining. (**B**) Graphical representation of the esophageal samples’ COX-2 staining score. Correlation analysis between Perilipin-2 and COX-2 percentage of positively stained cells (**C**) in the esophageal adenocarcinoma (EAC) and (**D**) in the esophageal squamous cell carcinoma (ESCC) samples. (**E**) Evaluation of LD (by using LipidTox) and COX-2 subcelullar localization, by immunofluorescence, in EAC (OE33) and in ESCC (OE21) cell lines. Zoom highlights LipidTox and COX-2 co-localization. Blue color represents DAPI staining; Red color represents LipidTox staining; Green color represents COX-2 staining. Experiments were performed in triplicate *p < 0.05; **p < 0.005.

COX-2 protein was evaluated by immunohistochemistry, in samples representing the evolution of EAC, as well as in ESCC samples. So, COX-2 was detected in 30% of the non-obese (NOE, with a median of 13.8% positive cells) and in 22% of the obese individuals esophageal samples (OE, with a median of 5.0% positive cells), as well as in 22% of BE patients (with a median of 35.0% positive cells) and in 100% of EAC patients (with a median of 43.0% positive cells), respectively. Finally, 66% of the ESCC samples were positive for COX-2, with a medium number of 29% positive cells. The inflammatory infiltrate (II) was also positively stained and used as the positive control (C+) (Fig. 4A,B).

Considering the percentage of COX-2 positive cells within the different groups, EAC samples displayed a significant increased number of COX-2 positive cells when compared to esophageal samples from non-obese and obese individuals (Fig. 4B). BE and ESCC groups also presented significant higher percentage of COX-2 positive cells, when compared to esophageal samples from obese individuals, whereas no significant differences were detected among BE, EAC and ESCC groups, although it is possible to observe a lower percentage of COX-2 positive cells in ESCC samples, when compared to BE and EAC samples (Fig. 4B). Further, correlation analyses were performed revealing a significant positive correlation (p < 0.0019; R^2^ = 0.3438) between PLIN2 and COX-2 expression in EAC samples (Fig. 4C), whether no significant correlation was detected in ESCC samples (p = 0.1769; R^2^ = 0.07781) (Fig. 4D).

In order to further investigate the correlation observed between PLIN2 and COX-2 staining in EAC samples, the subcellular localization of these two proteins was assessed by immunofluorescence in EAC and ESCC cell lines. Figure 4E shows that LD, selectively stained by LipidTox, and COX-2 are found co-localized in EAC cell line (OE-33), but not in ESCC cells (OE-21).

Collectively, these results suggest that LD presence may be associated with COX-2 production in EAC, but not in ESCC.

### BE and EAC risk factor associated stimuli are capable of inducing the expression and secretion of inflammatory mediators

**Figure 5:**
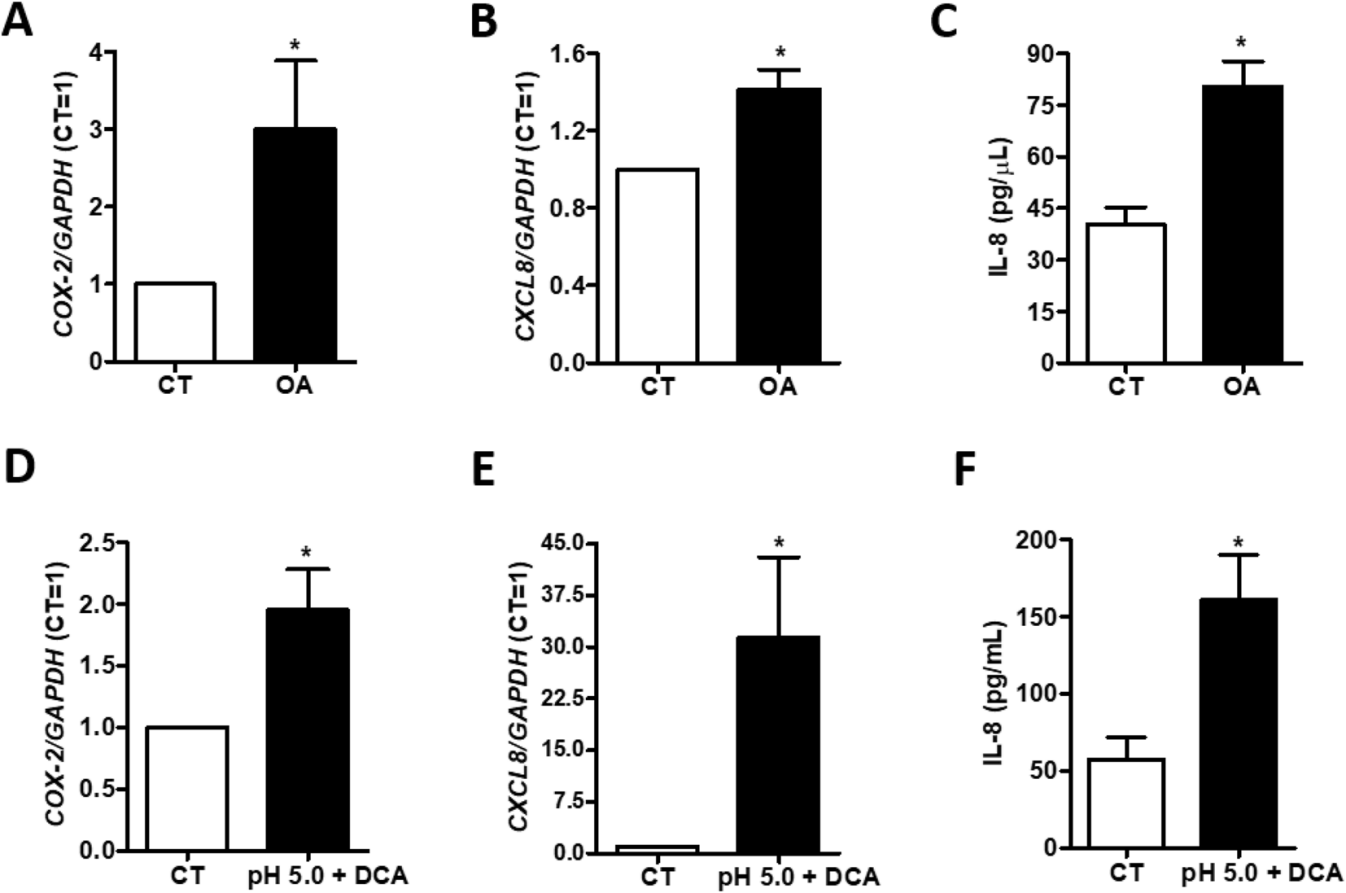
Evaluation of the expression and secretion of inflammatory mediators in the normal immortalized esophageal cell line, Het1a, upon exposure to esophageal adenocarcinoma (EAC) risk factors associated-stimuli. COX-2 (**A**, **D**) and CXCL8 (**B**, **E**) mRNA levels were assessed by RT-qPCR in, and IL-8 secretion by ELISA (**C**, **F**) in Het1a cells incubated with 5 µM of Oleic Acid (OA) for 24 h and stimulated with 5 pulses (3 min each) of acidified culture medium (pH 5.0) containing 200 μM of DCA. Experiments were performed in triplicate *p < 0.05.

*COX-2* and *CXCL8* expression, as well as Interleukin 8 (IL-8) secretion, were assessed by RT-qPCR and ELISA, respectively in Het1a cells incubated with 5 μM of OA for 24 h. OA treatment resulted in approximately 3.0- and 1.3-fold increase in *COX-2* and *CXCL8* mRNA levels, respectively, as well as in a 2.0-fold increase in IL-8 secretion in Het1a treated cells, when compared to control cells (Fig. 5A–C). Similarly, upon the combined treatment of 3 pulses of DCA 200 μM and acidified medium (pH5.0), Het1a cells displayed increased levels of *COX-2* and *CXCL8* mRNA of about 2.0- and 30-fold, respectively, compared to non-exposed cells. Additionally, the levels of secreted IL-8 were 3.0-fold greater in incubated, when compared to control cells (Fig. 5D–F).

These results suggest that the increased formation of LD entangled by the exposure to EAC associated risk factors may lead to an enhanced production of inflammatory mediators.

## Discussion

Obesity is one of the main risk factors associated with BE and EAC development^23^, and EAC is the tumor that harbors the highest relative risk of development associated to obesity^24^. Therefore, the initial increase in LD amount observed in esophageal tissue from obese individuals points out a connection between obesity and early esophageal cellular alterations that may predispose cells to other changes associated with BE and EAC development, since this increase is progressive throughout BE and EAC. Increased number of ectopic LD in obesity had already been reported, including in muscle, liver, and heart, where it has been associated to the pathogenesis of insulin resistance, type 2 diabetes, atherosclerosis, steatosis and nonalcoholic fatty liver disease^25–27^. Of note, this work was the first to demonstrate enhanced LD presence in esophageal tissue samples from obese individuals. Reinforcing the LD regulation throughout EAC development, an association between *PLIN-2* expression and the presence of dysplasia was detected in BE patients. It would had been interesting to further explore the association between the grade of dysplasia of BE patients and *PLIN-2* expression, to strengthen the progression model, nevertheless, due to the fact that BE samples analyzed were collected retrospectively, the information on dysplasia grade was not available, preventing us to perform this analysis. Here, it is important to stress out that, differently from the enhanced LD presence throughout the conditions representing EAC development, no significant changes were observed in ESCC tumors, when compared to non-tumor esophageal tissues, suggesting that it may not be a significant event for ESCC development.

Despite the complexity and multifactorial nature of obesity^28^ and GERD^29^, risk factors to BE and EAC, which is limiting to completely mimic these conditions, we employed OA and DCA and/or acidified culture medium treatments to simulate obesity and GERD in vitro. OA is detected in greater amounts in the blood of obese, when compared to non-obese individuals^30^. Further, OA is a powerful inductor of LD synthesis in different cell types, such as macrophages, epithelial cells, hepatic cells and leukocytes^31–33^. However, this is the first study to show this phenomenon in esophageal cells. The stimuli that mimic GERD, DCA and acid pulses, were also capable of inducing LD in esophageal cells, particularly when administered together to the cells. This is in accordance with other studies that showed that combination of bile acids and acid gastric juice presents a much greater potential to induce the development of BE and EAC, than either stimulus alone^34,35^.

OA is capable of inducing LD and *PLIN* through peroxisome proliferator activated receptors (PPAR), particularly PPARα^36,37^. *PLIN* genes code the most important proteins found on the surface of LD, coating it, and possessing a fundamental role in LD formation, maintenance, modification and involution^38^. Complementary, DCA is capable of inducing *COX2* expression through an interaction with PPARα and CREB (Cyclic AMP-Responsive Element-Binding Protein 1) that form a protein-DNA complex together with *COX-2* promoter, and, in turn, activate its transcription in human colorectal cancer cell lines^39,40^. Thus, we suggest that LD biogenesis enhancement and *PLIN2* induced expression reported in the present study in esophageal cells upon OA and DCA treatment may be due to the activation of PPARα signaling pathway. Nevertheless, further experiments should be performed to further explore the mechanisms through which EAC associated risk factors trigger *PLIN2* expression and LD biogenesis.

Additionally to the induction of LD biogenesis, BE and EAC risk factors associated-stimuli also triggered an induction of *CDX2 and CXCL8* expression, and IL-8 production in esophageal cells. CDX2 is a transcription factor that directs and maintains the differentiation of intestinal cells. Although it is not expressed in normal esophageal squamous epithelium, it is increased in non-dysplastic BE, indicating its presence as an early marker that leads to BE and EAC^41^. Moreover, CDX2 ectopic expression is sufficient to induce alterations associated with the intestinal phenotype in esophageal squamous epithelium^42^. The induction of *CDX2* expression by bile acids and acid-pH medium in esophageal cells had already been reported, both in murine and in vitro models^43–46^. However, enhancement of *CDX2* expression upon OA treatment had never been demonstrated in any cell type before. Complementary, enhanced IL-8 serum levels had already been reported in BE and EAC patients^21^, and in vitro models have demonstrated that DCA can induce *CXCL8* levels in esophageal cells^47,48^. These results together suggest that the transformation of esophageal epithelium and initiation of inflammation are tightly related and point out to a link between LD biogenesis and BE. It would had been interesting to further explore the link between LD’s biogenesis enhancement and EAC development, by using a BE cell line. The corroboration of our data in such model could strength our findings, nevertheless, we did not have access to this cell line, which represents a weakness of our study.

LD was present in EAC at significantly higher levels than in ESCC. The presence of LD had already been assessed in tumors originated in the same tissue, but from distinct histopathological subtypes, lung adenocarcinoma and squamous cell carcinoma, and no differences in LD levels were detected between these tumors^15^. This suggests that the greater amount of LD observed in EAC, when compared to ESCC, may not be associated with the histological difference between these two types of esophageal tumors, but rather by the risk factors that drive the carcinogenesis process for each specific tumor histotype. In fact, in vitro data demonstrated that the increment in LD amount, as well as in the expression of genes involved with its synthesis, *PLIN2* and *FASN*^18^, was triggered exclusively by the exposure of the esophageal epithelium to the risk factors associated with EAC development, once no association between ESCC-associated risk factors and LD generation was observed. LD was also present at much higher levels in EAC than in ESCC cell line, and this was followed by higher expression and secretion of inflammatory mediators, such as *COX-2*, *CXCL-8* and IL-8.

The use of nonsteroidal anti-inflammatory drugs reduces the risk of ESCC and EAC development^22^. COX-2 is the target of these drugs and has already been implicated in esophageal carcinogenesis, being upregulated in both tumors^49,50^. In this study, COX-2 expression was found progressively upregulated in the sample groups representing the evolution of EAC. Although COX-2 expression was also increased in ESCC samples, when compared to non-tumor samples, it was lower than in EAC. Further, there was a positive and significant correlation between PLIN2 and COX-2 expression in EAC, but not in ESCC, indicating that the induction of these two proteins may be co-regulated, as part of a common process related to EAC carcinogenesis. COX-2 staining was mainly cytoplasmic in the esophageal tissues assessed, in accordance to previous studies^51,52^, and perinuclear in some cases, which is considerably less frequent^53^. Most of COX-2 perinuclear staining was observed in ESCC samples. Further, LD and COX-2 were detected co-localized only within EAC cells, proposing that LD may be the site for eicosanoid production in this tumor. Thus, it could represent a potential difference in COX-2 inflammatory pathway between the two different esophageal tumor subtypes, regarding its activation and function.

The mechanisms through which these organelles contribute to tumorigenesis have not yet been fully elucidated. Nevertheless, a growing body of evidence suggest that enhanced LD amount may cope a wide range of stressful cellular conditions, by modulating lipid metabolism, providing this macromolecule for structural and energetics needs, as well as for inflammatory mediators production, enabling cancer cell survival and tumor growth^54^. In this sense, our group has recently demonstrated that cellular proliferation is based on LD meticulous regulation, pointing out an important link between cell cycle progression, cell proliferation and lipid accumulation^55^. Furthermore, lipid metabolism has been recognized as crucial for supporting cell proliferation^56^, through distinct mechanisms, such as mTOR activation by leptin signaling pathway^57^.

In conclusion, in this study we demonstrated that LD are increased along EAC evolution as a consequence of the exposure of the esophageal epithelium to the risk factors associated with BE and EAC. Moreover, the induction of LD generates an enhanced expression and secretion of inflammatory mediators, that may contribute to the establishment of these non-malignant conditions, thus leading to EAC development.

## Methods

All methods were carried out in accordance with relevant guidelines (Declaration of Helsinki).

### Patients and samples

Protein expression analysis comprised: 30 samples from Barrett’s esophagus (BE) patients who were submitted to endoscopy procedure at Hospital das Clínicas de Porto Alegre (HCPA/UFRGS), 25 esophageal squamous cell carcinomas (ESCC), and 64 esophageal adenocarcinoma (EAC) samples obtained from patients who had confirmed histopathology diagnosis and were submitted to surgery, between 2002 and 2010, at Brazilian National Cancer Institute (INCA) and the Hospital das Clínicas de Porto Alegre (HCPA / UFRGS). Additionally, ten esophageal samples from healthy non-obese individuals (NOE), and 21 from obese individuals (OE) who underwent endoscopy due to any reason other than cancer at Hospital Pedro Ernesto (HUPE/UERJ) were also included in this study. Samples were fixed in formalin and embedded in paraffin for further analysis. Epidemiological and clinicopathological data were obtained by using a standardized questionnaire, and from patients’ medical records. None of the patients comprised in this study had undergone any type of chemotherapy and/or radiotherapy before sample collection. The use of the human samples was approved by the Ethics Committee of institutions involved (INCA—115/10, HUPE/UERJ—416, HCPA/UFRGS—02 223). All patients and healthy individuals who kindly agreed to participate in the study signed an informed consent form.

### Immunohistochemistry

Immunohistochemistry (IHC) was performed on 4 μm paraffin sections of 10 NOE, 21 OE, 30 BE, 64 ADE, and 25 ESCC samples. For PLIN2 and COX-2 antigen retrieval, sections were incubated in water bath while submerged in a target buffer solution (DAKO), pH 6.0 and pH 9.0, respectively, for 40 min at 98 °C. Sections were then incubated with the primary antibodies anti-PLIN2 (AP125; Research Diagnosis Inc., working dilution 1:100), and anti-COX-2 (Cayman Chemicals, working dilution 1:200) during 12 h at 4 °C. The detection system used was the NovoLink Max Polymer Detection System (Leica Biosystems), following the protocol described by the manufacturer. Sections were counterstained with Harris’ hematoxylin. The staining positiveness evaluation was performed by three independent pathologists.

### Cell lines and treatments

OE-33 and OE-21 cell lines were derived from ADE and ESCC, respectively, and were obtained from ATCC. Both lineages were cultured in RPMI medium (Invitrogen) supplemented with 10% fetal bovine serum (Gibco) and 1% of the cocktail penicillin/glutamine/streptomycin (Invitrogen) and maintained at 37 °C, under 5% CO_2_. The immortalized normal esophageal epithelium cell line, Het1a, was also obtained from ATCC, and was cultured in BEBM plus supplements (Lonza) and maintained at 37 °C under 5% CO_2_. The three cell lines used in this study were authenticated by using STR (or SNP) profiling within the last 3 years and regularly tested for mycoplasma contamination, by using MycoSensor PCR Assay Kit (Agilent). Cells were stimulated with Oleic Acid (OA—1, 2 and 5 μM) for 24 h or untreated. Het1a cell line was incubated with deoxycholic bile acid (DCA—200 μM) or acidified medium (acid pH 5.0), or the combination of both, in plain medium at 37 °C under 5% CO_2,_ for variable number of 3 min pulses, with 30 min interval between them. Additionally, Het1a cells were treated with increasing amounts of nicotine-derived nitrosamine ketone (NNK—0.5, 1.0 and 2.0 μM) and increasing concentrations of ethanol (EtOH 0.01, 0.1 and 1.0%) for 24 h or untreated.

### Lipid droplets staining and enumerations

Cells (1 × 10^5^/well/6-well plate) were stimulated, or not, as described above, and stained for lipid droplets (LD) by using Oil Red O (ORO), since ORO selectively stains and detects neutral lipids. Briefly, cells were fixed in 3.7% formaldehyde (10 min), rinsed with PBS and incubated with 100% propylene glycol (5 min). Next, stained with ORO (Sigma) (10 min) pre heated at 60 °C, followed by 5 min incubation with 85% propylene glycol, then rinsed with PBS and incubated with DAPI (Molecular Probes-Invitrogen) (5 min). Photos of the stained cells were taken in a light microscope (Nikon, Tokyo, Japan) connected to a digital camera (Coolpix 990; Nikon). The number of LD per cell was determined by the software ImageQuant TL (GE Healthcare), using the option of colony counting.

### Immunofluorescence

3 × 10^4^ cells/well/were plated in a 24-well plate and, 24 h after, fixed with 3.7% formaldehyde, followed by permeabilization with PBS/saponin 0.05% and blockage with 2% bovine serum albumin (BSA). For PLIN2 and BODIPY double staining, cells were then incubated (2 h) with primary antibody anti-PLIN2 (AP125; Research Diagnosis Inc., working dilution 1:100), rinsed with PBS and then incubated with Alexa Fluor 568 anti-mouse (Life Technologies), together with BODIPY (Life Technologies), a neutral lipid marker, for 45 min. Next, several washes with PBS were performed, followed by incubation with DAPI (Molecular Probes, Invitrogen) for 5 min. For COX-2 and LipidTox double staining, cells were incubated overnight with primary antibody anti-COX-2 (SC-1747, Santa Cruz Biotechnology), and, then, washed several times in PBS and incubated with Dylight 488 donkey anti-goat (Ab98514, Abcam) for 1 h. Then, cells were stained with HCS LipidTOX Red neutral lipid stain (H34476, Thermo Fisher Scientific) for 30 min. Nuclei were stained with DAPI for 5 min. All slides were mounted with VECTASHIELD (Vector Laboratories). Slides were read in confocal fluorescence microscope Olympus BX60 (Olympus). Digital images were obtained using Fiji software.

### RNA extraction, reverse transcription and RT-qPCR

Trizol reagent was used to extract total RNA from tissues samples and cell lines, following manufacturer's instructions (Invitrogen). Extracted RNA samples were quantified by spectrophotometry and 1 μg of RNA from each sample was reversed transcribed by using SuperScript II Reverse Transcriptase (Invitrogen), according to manufacturer's protocol. *CDX2, CXCL-8,* and *COX-*2 expression analyses were performed in an Illumina Eco Real-Time PCR System (Illumina) using SYBR Green Master Mix (QiaGen) and 10 pmols of the oligonucleotides. *GAPDH* was evaluated as the endogenous control. The sequences of the specific oligonucleotides used follow: *COX-2* Forward: 5′ GCCCTTCCTCCTGTGCC 3′, *COX-2* Reverse: 5′ AATCAGGAAGCTGCTTTT TAC 3′; *CDX2* Forward: 5′ CAGCAGCTCTGCAGTACGTC 3′, *CDX2* Reverse: 5′ CTCAGGCCTTGGAAGAAGTG 3′; *CXCL8* Forward: 5′ CACCGGAAGGAACCATCT CACT 3′, *CXCL8* Reverse: 5′ TGCACCTTCACACAGAGCTGC 3′; GAPDH Forward: 5′ CAACAGCCTCAAGATCATCAGCAA 3′, GAPDH Reverse: 5′ AGTGATGGCATGGACTGTGGTCAT 3′. *PLIN2* and *FASN* gene expression was assessed by using Step One Plus Real-Time PCR System thermoycler, using PCR Mastermix TaqMan (Applied Biosystems). The TaqMan probe sets used were: *GAPDH* (Hs99999905_m1)—endogenous control—*FASN* (Hs00188012_m1) and *PLIN2* (Hs00605340_m1). The amplification reaction consisted in: 5 min for DNA pre-denaturation at 95 °C, followed by 40 cycles of hybridization and complementary chain synthesis for 5 s at 90 °C and 10 s at 60 °C. All the samples were analyzed in triplicate. Relative mRNA levels were determined by using the comparative threshold cycle (CT) with the analyzed gene expression levels normalized by those of *GAPDH* and using the not treated cell lines as the reference.

### IL-8 and PGE_2_ dosage

Cells (2 × 10^5^ per well) were stimulated, or not, as described above, for 48 h. Briefly, cells supernatant were collected, centrifuged, and then kept at − 20 °C for later use. Interleukin 8 (IL-8) and PGE_2_ were dosed using IL-8 EIA KIT-monoclonal (Cayman Chemicals) and PGE_2_ EIA KIT-Monoclonal (Cayman Chemicals), respectively, following the protocol described by the manufacturer. The result was measured in a spectrophotometer set to 420 nm. The result was given as pg/μL, relating the absorbance of the samples to a standard curve present in the kit.

### Analyses of *PLIN2* expression data deposited in The Cancer Genome Atlas

Both *PLIN2* expression and clinicopathological patients’ data from 95 ESCC and 89 EAC samples were downloaded from the open-access bio-database The cBioPortal for Cancer Genomics^58,59^, which allows the visualization and expression analysis of large-scale data sets of The Cancer Genome Atlas (TCGA).

### Statistical analysis

For continuous variables, the Kolmogorov–Smirnov test was applied to verify the Gaussian distribution of continuous data. For two groups analysis was used Mann–Whitney test. To analyze three or more groups ANOVA and Tukey post-test or Kruskal–Wallis test followed by Dunn's test were applied. Pearson correlation test was used to evaluate the correlation between COX-2 positive esophageal cells and Perilipin-2 positive cells. To assess the relationship between gene or protein expression levels and clinicopathological features, we used the Fisher's exact test. Statistical analyses were performed with GraphPad Prism 5.0 (GraphPad Software Incorporated, USA). The final values were considered of statistical significance when p < 0.05.

## Supplementary Information

Supplementary Information.

## Data Availability

All data generated or analyzed during this study are included in this published article [and its “Supplementary Information S1” files].
