# Supplementary material for: Lipid droplet biogenesis and COX-2 pathway activation are triggered by Barrett’s esophagus and adenocarcinoma, but not esophageal squamous cell carcinoma risk factors
Source: Sci Rep. 2021 Jan 13;11:981. doi: 10.1038/s41598-020-80035-4 (PMC7807011; doi:10.1038/s41598-020-80035-4)
Supplement: Supplementary file 1 — Supplementary Information. [file 41598_2020_80035_MOESM1_ESM.pdf]

Title

Lipid droplets biogenesis and COX-2 pathway activation are triggered by esophageal adenocarcinoma (EAC), but not esophageal squamous carcinoma (ESCC), associated risk factors

Carrossini, N.[1#]; Meireles Da Costa, N.[1#]; Andrade-Barreto E.[2]; Sousa, V. P. L.[1]; Nicolau-Neto, P.[1]; Souza-Santos, P.T. [1]; Mansur, G.R. [3]; Wernersbach, L. [4]; Bozza, P.T. [2]; Viola, J.P.B. [5]; Ribeiro Pinto, L.F.[1*]

[#]*Carrossini, N. and Meireles Da Costa, N. equally contributed to this work*

[*]*Corresponding author*

Authors and affiliations:

1. Molecular Carcinogenesis Program, Brazilian National Cancer Institute – INCA. Rua André Cavalcanti, 37 – 6º andar, Centro, Rio de Janeiro, RJ, Brasil. CEP: 20231-050.
2. Laboratory of Immunopharmacology, Oswaldo Cruz Institute - FIOCRUZ. Av. Brasil 4365, Manguinhos - Rio de Janeiro - RJ – Brasil. CEP.: 21040-360
3. Endoscopy Section, Brazilian National Cancer Institute – INCA. Praça Cruz Vermelha, 23 – 8º andar, Centro, Rio de Janeiro, RJ, Brasil. CEP.: 20230-130.
4. Pathology Division, Brazilian National Cancer Institute – INCA. Rua Cordeiro da Graça, 156, Santo Cristo, Rio de Janeiro, RJ, Brasil. CEP.: 20220-400.
5. Program of Immunology and Tumor Biology, Brazilian National Cancer Institute – INCA. Rua André Cavalcanti, 37 – 5º andar, Centro, Rio de Janeiro, RJ, Brasil. CEP: 20231-050.

Corresponding Author:

Dr. Luis Felipe Ribeiro Pinto

Head of the Molecular Carcinogenesis Program (PCM),

Research Coordination (CPQ),

Brazilian National Cancer Institute (INCA),

Rua André Cavalcanti, 37-Centro, 6º andar

Rio de Janeiro, CEP 20231-050 RJ, Brazil.

tel. +55 21 32076598

fax. +55 21 32076536

Supplementary Figure 1

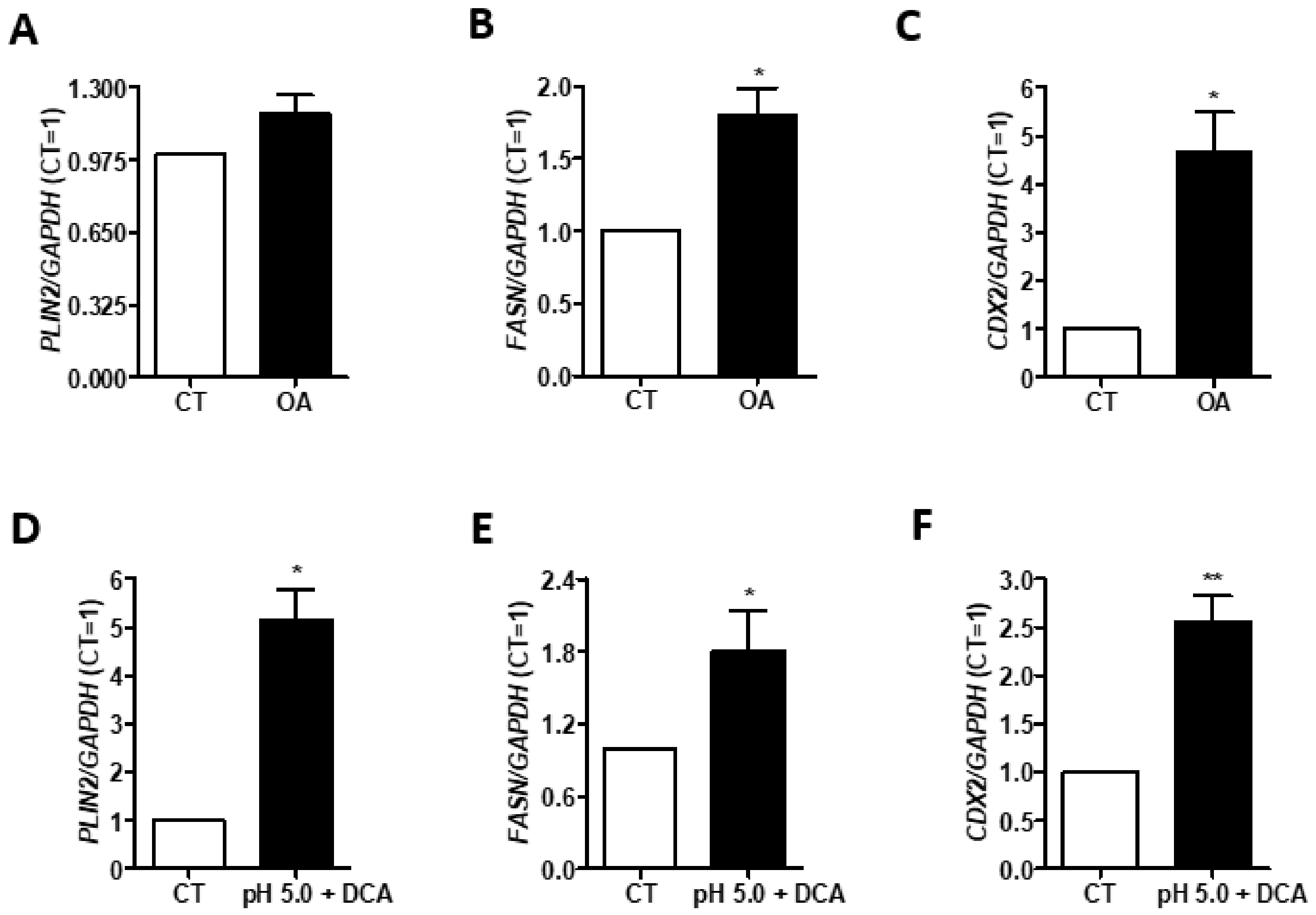

Supp Fig. 1 *PLIN2*, *FASN*, *CDX2* expression evaluation in the normal immortalized esophageal cell line, Het1a, upon exposure to esophageal adenocarcinoma (EAC) risk factors associated-stimuli. PLIN2 (A and D), FASN (B and E), CDX2 (C and F) mRNA levels were assessed by RT-qPCR in Het1a cells incubated with 5μM of Oleic Acid (OA) for 24h and stimulated with 5 pulses (3 min each) of acidified culture medium (pH 5.0) containing 200μM of DCA. Experiments were performed in triplicate *p<0.05; **p<0.005

Supplementary Figure 2

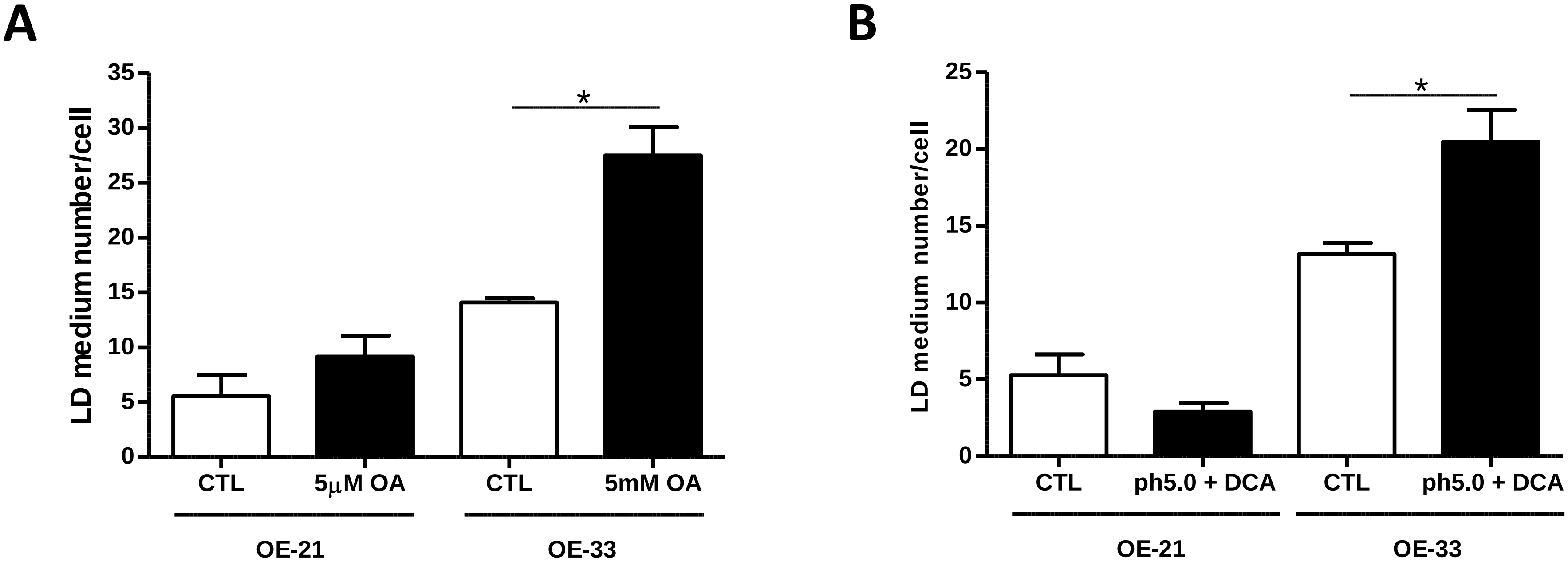

Supp Fig. 2 Evaluation of Lipid Droplets (LD) number in esophageal squamous cell (ESCC) and adenocarcinoma (EAC) cell lines, upon exposure to esophageal adenocarcinoma (EAC) risk factors associated-stimuli. LD quantification assessed by Oil Red O (ORO) staining in the esophageal squamous cell carcinoma (OE21) and adenocarcinoma (OE33) cell lines cells incubated with 5μM of Oleic Acid (OA) for 24h (A) and stimulated with 5 pulses (3 min each) of acidified culture medium (pH 5.0) containing 200μM of DCA (B). Experiments were performed in triplicate *$p<0.05$.

Supplementary Table 1: Clinicopathological characteristics of the individuals and patients comprised in the study

Characteristics	NOE (n=10)		OE (n=22)		BE (n=19)		EAC (n=59)		ESCC (n=25)	
Age										
Median (years)	55.0		34.5		64.0		62.0		56.0	
(Range)	(28 – 84)		(27 – 60)		(16 – 83)		(34 – 83)		(36 – 84)	
Follow-up										
Median (months)							12.13		31.56	
(Range)							(0.86 – 139.53)		(0.5 – 122.4)	
	n	%	n	%	n	%	n	%	n	%
Gender										
Female	4	40.0%	11	50.0%	10	52.6%	7	11.8%	6	24.0%
Male	6	60.0%	11	50.0%	9	47.3%	52	88.2%	19	76.0%
N/A	0		0		0		0		0	
Tobbacco Smoking										
No	7	70.0%	14	63.6%	4	21.0%	14	23.7%	5	20.0%
Yes	0	0.0%	2	9.1%	3	15.7%	20	33.8%	16	64.0%
Former	3	30.0%	6	27.3%	3	15.7%	24	40.6%	2	8.0%
N/A	0		0		9	47.3%	1	1.7%	2	8.0%
Alcohol Consumption										
No	2	20.0%	9	40.9%	6	31.6%	23	38.9%	14	56.0%
Yes	8	80.0%	13	59.1%	4	21.0%	21	35.6%	8	32.0%
N/A	0		0		9	47.3%	15	25.4%	3	12.0%
Tumor Localization										
Upper Third							0	0.0%	1	4.0%
Middle Third							10	16.9%	13	52.0%
Lower Third							37	62.7%	10	40.0%
N/A							12	20.3%	1	4.0%
Tumor Differentiation										
Well-differentiated							2	3.4%	2	8.0%
Moderately-differentiated							34	57.6%	16	64.0%
Poorly-differentiated							19	32.2%	5	20.0%
N/A							4	6.7%	2	8.0%
Tumor Stage										
I							3	5.1%	2	8.0%
II							11	18.6%	4	16.0%
III							16	27.1%	7	28.0%
IV							2	3.4%	4	16.0%
N/A							27	45.7%	8	32.0%
Gastroesophageal Reflux History										
No	7	70.0%	17	77.3%	63	66.3%				
Yes	3	30.0%	5	5.3%	14	14.7%				
N/A	0		0		18	18.9%				

N/A: not informed

NOE: non-obese individuals

OE: obese individuals

BE: Barrett's esophagus patients

EAC: esophageal adenocarcinoma patients

ESCC: esophageal squmous cell carcinoma patients

Supplementary Table 2: Association analysis between Perilipin-2 and COX2 staining and baseline characteristics of Barrett's esophagus (EB), esophageal adenocarcinoma (EAC) and esophageal squamous cell carcinoma (ESCC) patients investgated in the study.

	BE (n=19)		EAC (n=59)		ESCC (n=25)		BE (n=19)		EAC (n=59)		ESCC (n=25)	
**Characteristics**	**Perilipin-2 staining**						**COX2 staining**					
Age												
**r**	0.355		0.003		0.47		0.0456		0.1361		0.2743	
p	0.14		0.98		**0.02**		0.86		0.36		0.18	
	**Positive**	**Negative**	**Positive**	**Negative**	**Positive**	**Negative**	**Positive**	**Negative**	**Positive**	**Negative**	**Positive**	**Negative**
Gender												
**Female**	7	3	4	3	2	4	3	6	7	4	3	0
**Male**	6	3	34	18	8	11	5	5	27	21	18	4
p	1.00		0.69		1.00		0.64		0.68		1.00	
Tobbacco Smoking												
**Never**	3	1	8	6	3	3	1	2	6	4	3	2
**Yes**	6	0	29	15	6	12	3	4	27	21	14	5
**N/A**	9		1		1		9		1		1	
p	0.40		0.75		0.34		1.00		1.00		0.30	
Alcohol Consumption												
**Never**	5	1	9	14	4	4	2	3	12	5	5	3
**Yes**	4	0	23	13	5	9	1	4	21	21	10	4
**N/A**	9				3		9				3	
p	1.00		0.11		0.66		1.00		0.13		1.00	
Tumor Localization												
**Middle Third**			2	3	6	8			8	4	9	4
**Lower Third**			30	13	3	7			19	17	7	4
**N/A**			11		1				11		1	
p			0.32		0.68				0.50		0.77	
Tumor Differentiation												
**Well-differentiated**			1	1	1	1			0	2	2	1
**Moderately-differentiated**			24	12	7	9			23	14	7	10
**Poorly-differentiated**			12	7	1	4			10	8	2	3
**N/A**			2						2			
p			0.87		0.60				0.08		0.35	
Tumor Stage												
**Early**			11	3	2	4			7	4	6	0
**Late**			9	9	4	7			9	12	4	7
			17		8				17		8	
p			0.15		1.00				0.43		**0.034**	
Dysplasia												
**No**	6	0					5	5				
**Yes**	7	6					6	3				
p	**0.044**						0.62					

BE: Barrett's esophagus patients
EAC: esophageal adenocarcinoma patients
ESCC: esophageal squmous cell carcinoma patients
N/A: not informed

Supplementary Table 3: Clinicopathological characteristics of the esophageal adenocarcinoma (EAC) and esophageal squamous cell carcinoma (ESCC) patients from The Cancer Genome Atlas (TCGA) evaluated in the study

Characteristics	EAC (n=89)		ESCC (n=95)	
Age				
Median (years)	69.5		57.0	
(Range)	(27-86)		(36 – 90)	
Follow-up				
Median (months)	7.0		12.65	
(Range)	(0.36 – 83.18)		(0.3 – 67.97)	
	n	%	n	%
Gender				
Female	12	13.5%	14	14.7%
Male	76	85.4%	81	85.3%
N/A	1	1.1%	0	
Tobbacco Smoking				
No	24	27.0%	32	33.7%
Yes	8	9.0%	29	30.5%
Former	42	47.2%	30	31.6%
N/A	15	16.9%	4	4.2%
Alcohol Consumption				
No	27	30.3%	24	25.3%
Yes	60	67.4%	69	72.6%
N/A	2	2.2%	2	2.1%
Tumor Localization				
Upper Third	0	0,0%	6	6.3%
Middle Third	5	5.6%	44	46.3%
Lower Third	83	93.3%	44	46.3%
N/A	1	1.1%	1	1.1%
Tumor Differentiation				
Well-differentiated	3	3.4%	16	16.8%
Moderately-differentiated	28	31.5%	48	50.5%
Poorly-differentiated	27	30.3%	21	22.1%
N/A	31	34.8%	10	10.5%
Tumor Stage				
I	8	9.0%	21	22.1%
II	30	33.7%	38	40.0%
III	34	38.2%	28	29.5%
IV	11	12.4%	6	6.3%
N/A	6	6.7%	2	2.1%
Gastroesophageal Reflux History				
No	33	37.1%	63	66.3%
Yes	43	48.3%	14	14.7%
N/A	13	14.6%	18	18.9%
Esophagus Columnar Metaplasia				
No	29	32.6%	47	49.5%
Yes	31	34.8%	1	1.1%
N/A	29	32.6%	47	49.5%

N/A: not informed

EAC: esophageal adenocarcinoma patients

ESCC: esophageal squmous cell carcinoma patients

Supplementary Table 4: Association analysis between *PLIN2* expression and baseline characteristics of esophageal adenocarcinoma (EAC) and esophageal squamous cell carcinoma (ESCC) patients from The Cancer Genoma Atlas (TCGA) investigated in the study.

	PLIN2	
	EAC (n=89)	ESCC (n=95)
Characteristics		
Age (years)		
r	-0,01	-0.11
p	0.922	0.288
Gender		
Female	825.0	141.5
Male	870.6	392.9
p	0.571	**0.0078**
Tobbacco Smoking		
No	1034.0	1034.0
Yes	810.1	810.1
p	0.19	0.19
Alcohol Consumption		
No	874.7	437.0
Yes	810.1	376.1
p	0.786	0.4528
Tumor Localization		
Upper Third	0	519.1
Middle Third	1283.0	338,9
Lower Third	815.6	357,8
p	0.1	0.528
Tumor Differentiation		
Well-differentiated	671.4	397.2
Moderately-differentiatec	867.4	335.1
Poorly-differentiated	994.2	648.2
p	0.215	0.215
Tumor Stage		
Early (I + II)	660.3	320.6
Late (III+IV)	896.4	429.7
p	0.062	0.352
Esophagus Columnar Metaplasia		
No	796.4	
Yes	896.4	
p	0.756	
Gastroesophageal Reflux History		
No	854.2	
Yes	866.4	
p	0.966	

PLIN2 expression levels are expressed in RSEM+1

EAC: esophageal adenocarcinoma patients

ESCC: esophageal squmous cell carcinoma patients
